# Alternatives to free flap surgery for maxillofacial reconstruction: focus on the submental island flap and the pectoralis major myocutaneous flap

**DOI:** 10.1186/s12903-021-01563-7

**Published:** 2021-04-19

**Authors:** J. K. Meier, S. Spoerl, G. Spanier, M. Wunschel, M. J. Gottsauner, J. Schuderer, T. E. Reichert, T. Ettl

**Affiliations:** Department of Cranio-Maxillofacial Surgery, University Medical Center Regensburg, 93042 Regensburg, Germany

**Keywords:** Maxillofacial reconstructive surgery, Charlson comorbidity index, CCI, Microvascular, Flap surgery, Head and neck surgery

## Abstract

**Background:**

Microvascular tissue transfer (MTT) has been established as the gold standard in oral- and maxillofacial reconstruction. However, free flap surgery may be critical in multimorbid elderly patients and after surgery or radiotherapy, which aggravate microsurgery. This study evaluates indications and outcome of the submental island flap (SMIF) and the pectoralis major myocutaneous flap (PMMF) as alternatives to the free radial forearm flap (RFF).

**Methods:**

This retrospective study included 134 patients who had undergone resection and reconstruction with SMIF, PMMF, or RFF at our department between 2005 and 2020. The level of comorbidity was measured with the *Age-adjusted Charlson comorbidity index (ACCI).* Primary outcome variables were flap success, complications, wound dehiscence, surgery duration, as well as time at the ICU and the ward (hospitalization). Chi-square tests, t-tests, and ANOVA were performed for statistics.

**Results:**

24 SMIFs, 52 RFFs, and 58 PMMFs were included in this study. The flap types did not significantly differ in terms of flap success, complications, and healing disorders. The SMIF presented a success rate of 95.8% and was significantly more often used in elderly patients (mean age = 70.2 years; *p* < 0.001) with increased comorbidities than the PMMF (*p* < 0.01) and RFF (*p* < 0.001). SMIF reconstruction reduced surgery duration (*p* < 0.001) and time at the ICU (*p* = 0.009) and the ward (*p* < 0.001) more than PMMF and RFF reconstructions. PMMF reconstruction was successful in 91.4% of patients and was more frequently used after head and neck surgery (*p* < 0.001) and radiotherapy (*p* < 0.001) than SMIF and RFF reconstructions. Patients undergoing PMMF reconstruction more frequently required segmental jaw resection and had presented with advanced tumor stages (both *p* < 0.001). Nicotine and alcohol abuse was more frequent in the RFF and PMMF groups (both *p* < 0.001) than in the SMIF group.

**Conclusions:**

The pedicled SMIF represents a valuable reconstructive option for elderly patients with increased comorbidity because of the shorter duration of surgery and hospitalization. On the other hand, the PMMF serves as a solid backup solution after head and neck surgery or radiotherapy. The rates of flap success, complications, and healing disorders of both pedicled flaps are comparable to those of free flap reconstruction.

## Introduction

In the past decades, microvascular tissue transfer (MTT) has become more and more important in complex oral and maxillofacial reconstructions and is now considered the *gold standard* [1].

In spite of several advantages, microvascular free flap surgery has some drawbacks, such as extended operating time, increased requirement of personal, material, and financial resources, as well as the potential risk of anastomosis failure [2]. Such failure may become critical in comorbid elderly patients who require short operating times. In patients with previous head and neck surgery or radiotherapy (RT), microsurgery may be difficult due to scarring and vessel-depleted necks [3]. For such patients, pedicled flaps such as the pectoralis major myocutaneous flap (PMMF) [4, 5] or the submental island flap (SMIF) [6] are possible alternatives for oral and maxillofacial reconstruction. This work evaluates the indications and outcome of the SMIF and the PMMF at our institute in comparison to the radial forearm flap (RFF) as the most frequently used method in microvascular maxillofacial reconstruction.

## Materials and methods

### Patients

This retrospective study included 134 patients who had received reconstructive surgery with an SMIF, PMMF, or RFF in the oral and maxillofacial region at the Department of Cranio-Maxillofacial Surgery of the University Medical Center Regensburg between 2005 and 2020. During this period, the most frequently used flap at our department was the PMMF; the SMIF has been implemented since 2017, and the RFF was used between 2013 and 2015. Patient data were retrieved from the charts and the electronic patient data management system. The study was approved by the Institutional Ethics Committee (Approval No. 14–101-0095). Investigated outcome parameters were flap success, complication rates, revision surgery, healing disorders, flap necrosis, donor site morbidity, incision to suture time (total surgery time), stay at the intensive care unit (ICU), and stay on the ward. Flap success was defined as working transplant without any signs of transplant loss up to 6 weeks after surgery.

**Fig. 1:**
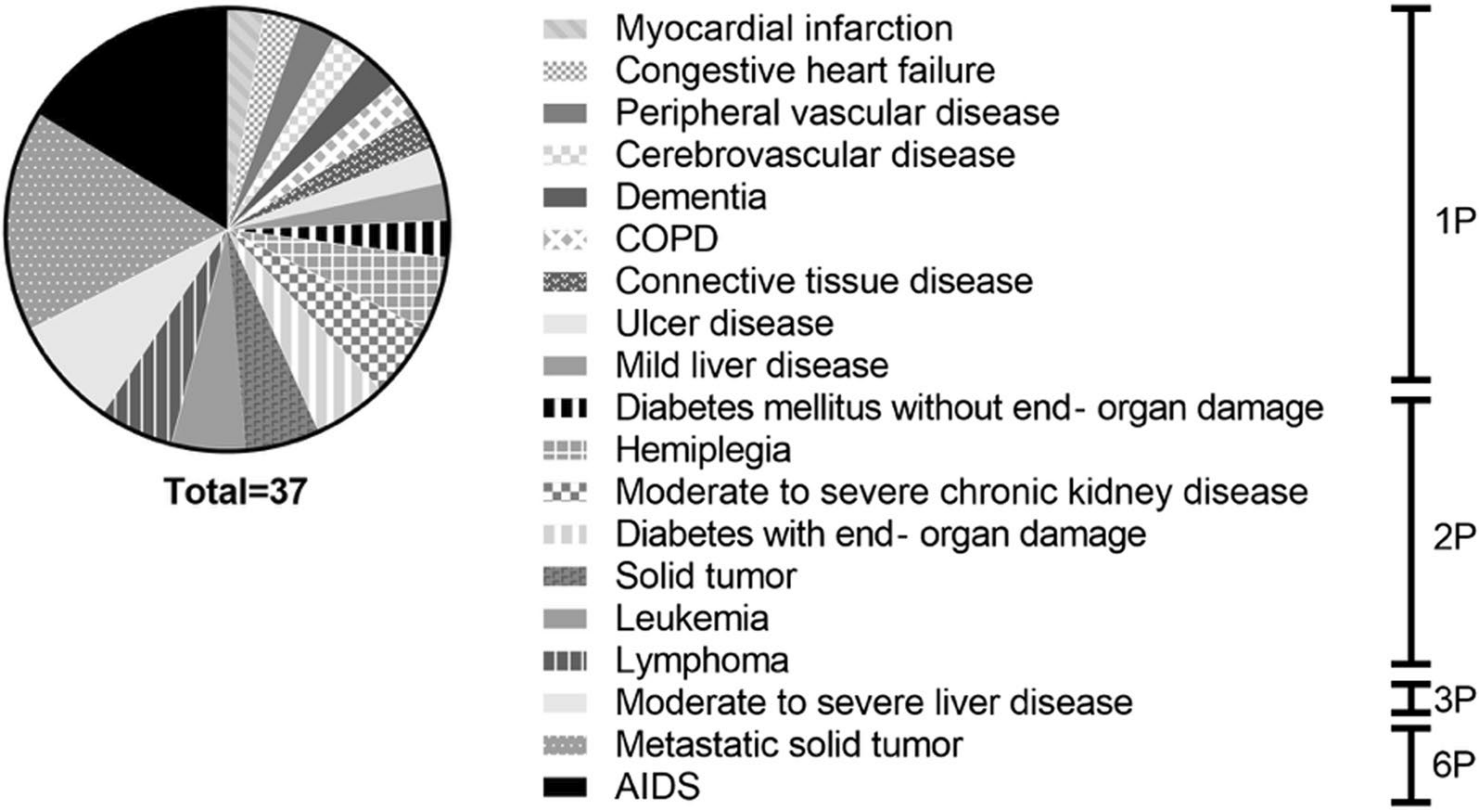
Charlson comorbidity index; incorporated diseases, weighting range from 1 to 6 points. To adjust for age, every decade after 40 years is reflected by 1 point (maximum weight for age: 4 points)

**Fig. 2:**
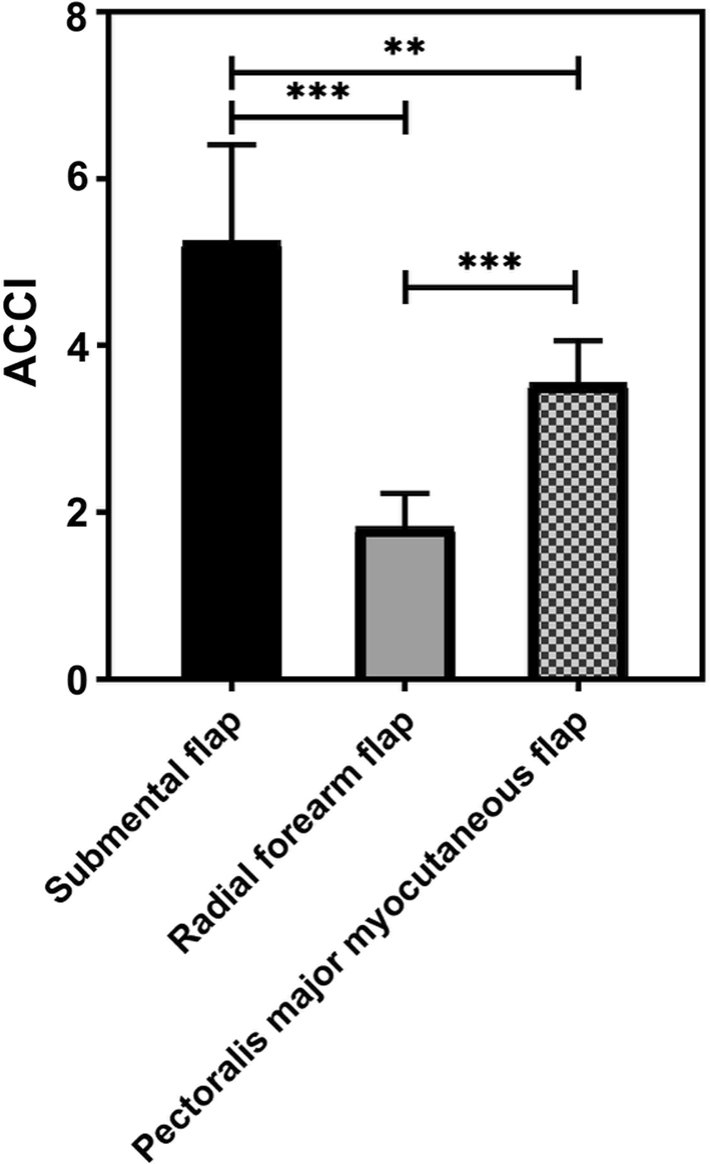
Age-adjusted Charlson comorbidity index for distinct flap types; *p-value < 0.05; **p-value < 0.01; ***p-value < 0.001

The level of comorbidity was measured with the *Age-adjusted Charlson comorbidity index (ACCI)* [7], and patients were classified according to the score (Figs. 1, 2). In order to evaluate patient-specific indications for the three flaps, flap type was associated with the ACCI.

**Fig. 3:**
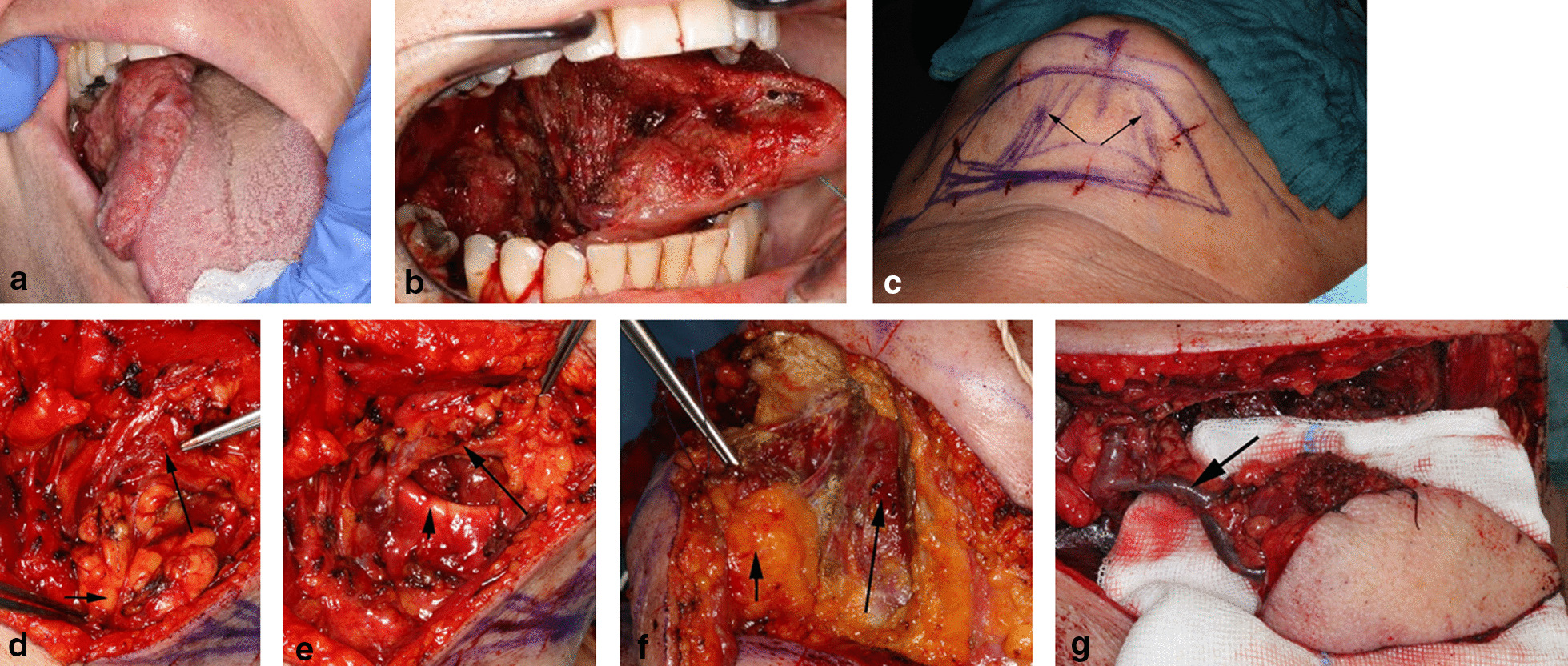
Preparation of the submental island flap; **a** Extended oral squamous cell carcinoma at the right side of the tongue; **b** Need for reconstruction after R0 resection (hemiglossectomy); **c** Marked submental island flap (7 × 5 cm); Anterior belly of digastric muscle on both sides (arrows). **d** Sub-mental vessels (long arrow) with retracted submandibular gland (short arrow); **e** Submental vessels (long arrow) after resection of the gland; tendon of digastric muscle (short arrow) **f** Harvested flap with included anterior belly of ipsilateral digastric muscle (forceps) and attached level 1a lymph nodes (short arrow) before dissection from the perforator; contralateral digastric anterior muscle in situ (long arrow) attached to the mandible **g** Submental island flap pedicled on the submental vessels (arrow)

**Fig. 4:**
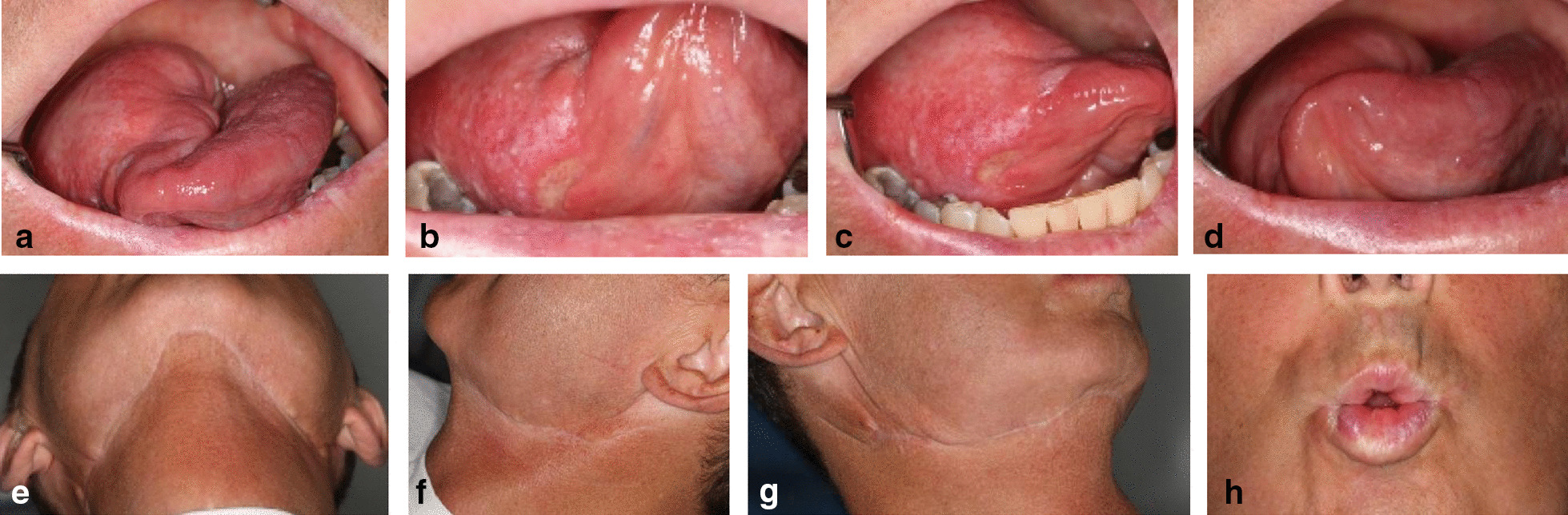
Post-surgical outcome; **a–d** Reconstructed tongue 6 months after surgery with excellent function and mobility; **e–g** Cervical scar after harvesting the submental island flap from the right side, neck dissection on both sides and adjuvant RT; **h** Preserved function of the marginal branch of the facial nerve

The RFF and the PMMF were harvested as extensively described in the literature [8–10]. The SMIF was raised as follows:

The patient is positioned in extended supine position, and the required flap size is marked in elliptical shape 1 cm below the inferior border of the mandible. The flap is centered over the ipsilateral anterior belly of the digastric muscle with its perforators. Skin laxity is checked by means of a pinch test to achieve primary closure without any tension (incisions up to 6 cm in width are easy to close). The upper incision runs through the skin, the subcutaneous tissue, and the platysma 1 cm below the mandibular border measured from the mandibular angle to the chin. First, the facial vein comes into view at the middle portion of the submandibular gland, which is removed to access the submandibular groove. Here, the submandibular vessels and the cranially running marginal branch of the facial nerve come into view. The nerve is preserved, and the preparation runs on the mylohyoid muscle in anterior direction, clipping small branches, and dissecting the adjacent lymph nodes at level 1b. Reaching the osseous fixation of the anterior belly of the digastric muscle at the chin, the muscle fibers are cut off, and the harvested muscle belly is sutured to the flap skin to prevent shearing of the perforators. Now the flap is circumcised, and the tendon of the anterior digastric muscle is cut off from the hyoid bone. Finally, the adjacent lymph nodes at level 1a are carefully dissected, preserving the perforators. If more flexibility of the flap is needed, the vascular pedicle is dissected down to the origin of the facial artery (at the upper border of the posterior belly of the digastric muscle) and the facial vein (down to the internal jugular vein). The flap is temporarily reattached to the cranial skin flap, which allows completion of the neck dissection procedure. For intraoral reconstruction, the flap is passed behind the mylohyoid muscle into the oral cavity. Reconstruction of facial defects requires a wide subcutaneous tunnel to minimize pressure on the pedicle at the angle of the mandible. A subcutaneous passive drain prevents compression by hematoma. The donor site defect is closed by advancing the subplatysmal-undermined inferior skin flap, and excess skin is removed according to Burow. A clinical case with intra- as well as postoperative photo-documentation is shown in Figs. 3 and 4.

### Statistics

For statistical analysis, SPSS (v. 25) was used, and a *p*-value ≤ 0.05 was defined as statistically significant. For univariate analysis, chi-square tests were used to compare different groups of outcome parameters. The t-test (two-sided) was used for comparison of mean values. One-way ANOVA was performed in the case of more than two means.

## Results

Table 1 comprises general patient data including specific information on associations between distinct flap types and clinicopathological factors. The study included more male (n = 98) than female (n = 36) patients, mean age was 63.3 (range 33–94) years. Overall, 24 patients had received an SMIF, 52 an RFF, and 58 a PMMF. In elderly patients, SMIFs (mean age = 70.2 years) were more often used than RFFs (57.6 years) and PMMFs (62.6 years) (*p* < 0.001). 119 (83.2%) patients required reconstruction for defects after ablative tumor surgery (all SMIFs and RFFs). In the PMMF group, 22.4% of flaps were required because of osteoradionecrosis (*p* < 0.001). 67.9% of all patients had a history of nicotine abuse, 70.1% in the PMMF group, 78.8% in the RFF group, and 37.5% in the SMIF group (*p* = 0.001). Additionally, alcohol abuse was significantly more prevalent in the RFF (69.2%) and PMMF (53.4%) group than in the SMIF (20.8%) group (*p* < 0.001). Patients in the PMMF group had more frequently a history of head and neck surgery (69.0%) and RT (55.2%) than patients in the SMIF (25.0% for surgery and 0% for RT) group and the RFF (11.5% for surgery and 2.0% for RT) group (*p* < 0.001 both). Regarding the recipient flap site, the SMIF was used for different intra- and extra-oral sites whereas the RFF was predominantly used for tongue and floor of mouth reconstructions (73.1%). The PMMF was frequently chosen for mandible (48.3%) as well as tongue and floor of mouth (36.2%) reconstructions. The PMMF (56.9%) was also more often used than the SMIF (12.5%) and the RFF (1.9%) in the case of jaw resections, particularly for segmental mandibular resections. With regard to the tumor (T)-stage classification of malignancies, the PMMF was used for patients with more advanced stages (64.0% for T3/T4) followed by the SMIF (47.6%). In contrast, only 11.5% of the patients with an RFF had been diagnosed with a higher T-stage (*p* < 0.001). Positive cervical lymph nodes (pN +) were more often found in the PMMF (38.5%) and RFF (44.2%) groups than in the SMIF (10.0%) group (*p* = 0.024).

Major perioperative complications occurred in 18 (13.4%) patients; 13 (72.2%) patients developed venous congestions, 6 of them due to hematoma. In 4 patients, arterial impairment led to severe perioperative complications, and 1 patient additionally developed serious wound infection. However, no significant association was found between complications and the type of flap. History of nicotine abuse (16.5% [yes] vs 7.0% [no]) as well as prior surgery in the head and neck (19.2% [yes] vs 9.7% [no]) involved a higher risk of perioperative complications. Wound healing disorders were highest in the PMMF (29.3%) group followed by the RFF (23.1%) and the SMIF (8.3%) group. Impaired wound healing was significantly associated with jaw resection (*p* = 0.012) and was non-significantly higher in patients with previous surgery (30.7% [yes] vs 20.7% [no]) or advanced T-stages (35.7% for T3/T4 vs 19.7% for T1/T2). Overall flap success was 92.5%: 95.8% for SMIFs, 92.3% for RFFs, and 91.4% for PMMFs. Flap failure was associated with previous surgery (15.4% vs 2.4%, *p* = 0.014). Increased comorbidity (ACCI > 3) did not correlate with flap complications, impaired wound healing, and flap success.

Table 2 presents associations between the type of flap and treatment-specific factors. Mean flap size was larger in the PMMF (67.4 mm^2^) group than in the RFF (31.1 mm^2^) and SMIF (37.0 mm^2^) group (*p* = 0.008). Mean duration of surgery, time at the ICU, and time on the ward (hospitalization) were significantly shorter for SMIF (297.5 min, 1.5 d, 12.09 d) reconstructions than for RFF (484.5 min, 4.3 d, 14.4 d) and PMMF (393.8 min, 3.4 d, 19.2 d) reconstructions (*p* < 0.01).

Figure 2 shows associations between the used flaps and patient comorbidity. Patients in the SMIF (mean score 5.25) group showed the highest degree of comorbidities followed by patients in the PMMF (3.55) and the RFF (1.8) group (*p* < 0.01).

## Discussion

Free flap reconstruction currently represents the first choice in managing complex maxillofacial defects because their functional and esthetic results and donor site morbidity rates are better than those of pedicled flaps [11]. Free flaps, which can also be successfully used in elderly patients, also have similar transplant survival rates [3, 12]. However, several publications have shown that perioperative local and systemic complications directly correlate with increased age and particularly with the presence of relevant comorbidities [13]. Other authors have stated that older patients are less likely to be discharged into their own home at the end of hospitalization. Age and length of stay have been identified as risk factors that necessitate discharge into a nursing facility after microvascular reconstruction [14]. Besides age and length of stay, increased comorbidities—identified by means of the ASA (American Society of Anesthesiologists) classification or the ACCI—influence the risk of perioperative complications and subsequently discharge into nursing rehabilitation [14]. In a French study evaluating free flap reconstructions in patients aged more than 70 years, the Charlson comorbidity index (CCI) was significantly associated with the length of hospital stay [15]. In a prospective study by Rosenberg et al., increased ASA scores and CCI correlated significantly with severe medical complications after microvascular tissue transfer in the head and neck region [16].

As described recently, free flap reconstruction represents the state of the art procedure at our department, and patients undergoing this procedure are of advanced age [3]. However, we have observed that the age at which patients require advanced maxillofacial flap reconstruction is increasing; thus, patients often present with a higher number of comorbidities. For these patients, the SMIF has become a reliable alternative to free flap reconstruction, particularly because of its shorter duration of both surgery and hospitalization. Besides the SMIF, another pedicled backup flap has been frequently used at our department: The PMMF has shown reliable results in patients with a history of RT, thus providing a valid option for treating even extensive defects after oncologic surgery.

Aim of the current study was to evaluate indications and outcome of different transplant options in reconstructive surgery. Hereby, the SMIF and the PMMF were compared with the RFF as the most frequently used microvascular flap.

Our results strikingly underline that the SMIF is the first reconstructive choice for patients presenting with severe comorbidities according to the ACCI.

The CCI, which has been cited in the literature over 8800 times [17], represents a manifold validated model for assessing comorbidities [18]. Especially in head and neck cancer, the CCI has been proven as a valid prognostic indicator for outcome of patients with cancer [19]. However, its applicability in reconstructive head and neck surgery may demand some modifications: The vascular status of patients, which is currently not reflected in the CCI, plays a key role in free flap surgery in the head and neck region. In a comprehensive cohort study, smoking and previous radiation therapy in the head and neck dramatically impaired flap outcome due to vascular complications [3]. Adapting the CCI to today's requirements in head and neck surgery, such clinical scoring systems will therefore certainly provide accurate tools for predicting mortality or adjusting for confounding in future cohort studies [20].

Since its first description by Martin et al. in 1990 [6, 21], the SMIF has been a widely used pedicled alternative in head and neck reconstruction. The SMIF provides reliable perfusion of the submental skin as well as constant vascular anatomy in the submandibular space on the mylohyoid muscle [22]. Faltaous et al. demonstrated in their anatomical studies that, in 70% of cases, the submental vessels run deep in the anterior belly of the digastric muscle. In only 30% of cases, the submental vessels run superficially to the muscle and sometimes even through the muscle [23]. The flap has a long-reaching (up to 8 cm), reliable pedicle, and cutaneous dimensions can be extended up to 7 × 18 cm. The SMIF may be used as a cutaneous, musculofascial (cervicofascial and platysmal) or an osteocutaneous flap. This flap has an excellent skin color match as well as a wide arc of rotation that allows extension to the entire ipsilateral face (except for a part of the forehead) and the whole oral cavity [21]. Corresponding to our results of SMIF reconstruction, other authors have also reported decreased surgery time, shorter hospitalization, fewer perioperative complications, and disease recurrence rates potentially similar to those of free tissue transfers for reconstructing oral cavity defects [24]. In addition, SMIF reconstruction is associated with lower postoperative costs and shorter ICU stays than microvascular reconstruction [25].

The comparison of the SMIF with the RFF was particularly impressive because it showed much shorter hospital stays of patients after SMIF reconstruction (Forner, Phillips, and Rigby). Some authors additionally evaluated the necessity of ICU surveillance after RFF and SMIF surgery. Hereby, just 1 patient required one night at the ICU after SMIF surgery compared to a mean ICU stay of 4.7 days in the RFF group. Underlining these observations, Paydarfar et. al. have even argued for a broad indication of the SMIF: For oral cavity defects measuring less than 40 cm^2^, the SMIF should be the reconstructive option of choice in defect areas suitable for the use of this type of flap (Paydarfar, Patel 2011).

After SMIF reconstruction, donor sites can be primarily closed with very good esthetic results [26]. Reported rates of complications and healing disorders are low with partial necrosis described in 5.1% of patients, complete necrosis in 1.7%, and reversible lesions of the marginal mandibular branch of the facial nerve in 1.1% [27]. SMIF reconstruction after tongue cancer preserves the ability of speech and swallowing, comparable to the results of microvascular reconstruction [28].

To increase the efficiency and reliability of the SMIF, Zenga et. al. recommended manual blunt dissection of the mylohyoid muscle and its inclusion into the submental island flap [29]. This technique requires meticulous dissection to avoid lymph node-bearing tissue residue at level I [30, 31]. Previous RT is no contraindication for the SMIF but certainly compromises wound healing in the submental donor area [32]. Our subset of SMIFs did not include a single patient with previous RT, and only a minority of patients had a history of head and neck surgery. However, one relevant disadvantage of pedicled SMIFs is that they are not an option after resection of the submental vessels, for instance during previous neck dissection.

Previous RT and surgery aggravate microvascular head and neck reconstruction with free flaps due to the impaired anatomy of the recipient vessels and reduced wound healing. In our study, PMMFs were predominantly used in patients with previous head and neck surgery and RT in contrast to microvascular flap entities such as the RFF. Moreover, patients in our PMMF group often had a history of both nicotine and alcohol abuse that was comparable to that in the RFF group but significantly higher than that in the SMIF group. Additionally, the PMMF was preferentially used for more voluminous defects after segmental jaw resection and alloplastic reconstruction requiring more bulky muscular tissue. Therefore, we used the PMMF in patients with major defects and uncertain vascular conditions, which was in line with current recommendations [5]. The main problem of using PMMF for reconstructive surgery was wound healing disorders, which seems reasonable in this specific group of patients who have a higher risk of developing adverse events than patients with an SMIF because of the widespread prevalence of nicotine abuse or previous RT. In the literature, the most frequently reported complication is partial or total flap necrosis during the development of the flap, often in association with previous RT [33]. Nevertheless, PMMF reconstruction resulted in an overall flap success rate of 91.4%, which is, in our eyes, a very satisfactory outcome in this distinct group of compromised patients.

## Conclusions

The submental island flap as well as the pectoralis major myocutaneous flap represent valuable alternatives to free flap reconstruction and have a similar outcome to free radial forearm flap reconstructions in terms of flap success, complication rates, and wound healing disorders. This work focused on the SMIF with its broad scope of application and showed that SMIF represents a clinically relevant alternative to microvascular tissue transfer for defects in the oral cavity. Additionally, the SMIF has been identified as a reliable reconstructive tool for older patients with significant comorbidities who will benefit from reduced duration of surgery and hospitalization. On the other hand, the pectoralis major flap remains an important back-up flap for patients after RT or repeated surgery who require larger amounts of musculocutaneous tissue.

**Table 1:** Flap type, clinicopathological factors, and outcome

	Flap type				
	SMIF	RFF	PMMF	Total	*p*-value
Age					
Mean	70.2	57.6	64.0	62.6	< 0.001
Sex					n.s
Male	16	37	45	98	
Female	8	15	13	36	
Nicotine					0.001
No	15	11	17	43	
Yes	9	41	41	91	
Alcohol					< 0.001
No	19	16	27	62	
Yes	5	36	31	72	
Prior surgery					< 0.001
No	18	46	18	82	
Yes	6	6	40	52	
Prior radiation therapy					< 0.001
No	24	51	26	101	
Yes	0	1	32	33	
Site					
Mandible/FO M	8	3	28	39	
Maxilla	1	3	2	6	
Tongue/FOM	4	38	21	63	
Cheek	6	8	1	15	
Extra-oral	5	0	6	11	
Jaw resection					< 0.001
No	16	27	14	57	
Partial	5	24	11	40	
Segmental	3	1	33	37	
Neck dissection					n.s
No	3	3	5	11	
Yes	21	49	53	123	
Tracheostomy					n.s
No	18	23	37	78	
Yes	6	28	21	55	
T-stage					< 0.001
T1–T2	11	46	14	71	
T3–T4	10	6	26	42	
N-stage					0.024
N-neg	18	29	24	71	
N-pos	2	23	15	40	
Complications					n.s
No	21	45	50	116	
Yes	3	7	8	18	
Healing disorders					n.s
No	22	40	39	101	
Yes	2	12	19	33	
Flap success					n.s
No	1	4	5	10	
Yes	23	48	53	124

**Table 2:** Flap type and treatment-specific factors

	Flap type				
	SMIF	RFF	PMMF	Total	*p*-value
Size					0.008
Mean (mm^2^)	37.0	31.1	67.4	43.9	
Surgery time					< 0.001
Mean (min)	297.5	484.5	370.4	393.8	
Time at ICU					0.009
Mean (days)	1.5	4.3	3.2	3.4	
Time at ward					< 0.001
Mean (days)	12.9	14.4	23.8	19.2	

## Data Availability

The datasets generated and analyzed during the current study are not publicly available because of restrictions in terms of data protection that are fixed in the given treatment contract by each patient and the approval of the local Ethics Committee. However, upon reasonable request to the corresponding author, we would consider making the data available in anonymized form.

## Abbreviations

ASA: American Society of Anesthesiologists
ACCI: Age-adjusted Charlson comorbidity index
CCI: Charlson comorbidity index
ICU: Intensive care unit
MTT: Microvascular tissue transfer
PMMF: Pectoralis major myocutaneous flap
RFF: Radial forearm flap
RT: Radiotherapy
SMIF: Submental island flap
